# A differential expression of pyrethroid resistance genes in the malaria vector *Anopheles funestus* across Uganda is associated with patterns of gene flow

**DOI:** 10.1371/journal.pone.0240743

**Published:** 2020-11-10

**Authors:** Maurice Marcel Sandeu, Charles Mulamba, Gareth D. Weedall, Charles S. Wondji

**Affiliations:** 1 Department of Medical Entomology, Centre for Research in Infectious Diseases (CRID), LSTM Research Unit, Yaoundé, Cameroon; 2 Department of Microbiology and Infectious Diseases, School of Veterinary Medicine and Sciences, University of Ngaoundéré, Ngaoundéré, Cameroon; 3 Department of Vector Biology, Liverpool School of Tropical Medicine, Liverpool, United Kingdom; 4 Uganda Virus Research Institute, Entebbe, Uganda; National Cheng Kung University, TAIWAN

## Abstract

**Background:**

Insecticide resistance is challenging the effectiveness of insecticide-based control interventions to reduce malaria burden in Africa. Understanding the molecular basis of insecticides resistance and patterns of gene flow in major malaria vectors such as *Anopheles funestus* are important steps for designing effective resistance management strategies. Here, we investigated the association between patterns of genetic structure and expression profiles of genes involved in the pyrethroid resistance in *An*. *funestus* across Uganda and neighboring Kenya.

**Methods:**

Blood-fed mosquitoes *An*. *funestus* were collected across the four localities in Uganda and neighboring Kenya. A Microarray-based genome-wide transcription analysis was performed to identify the set of genes associated with permethrin resistance. 17 microsatellites markers were genotyped and used to establish patterns of genetic differentiation.

**Results:**

Microarray-based genome-wide transcription profiling of pyrethroid resistance in four locations across Uganda (Arua, Bulambuli, Lira, and Tororo) and Kenya (Kisumu) revealed that resistance was mainly driven by metabolic resistance. The most commonly up-regulated genes in pyrethroid resistance mosquitoes include cytochrome P450s (*CYP9K1*, *CYP6M7*, *CYP4H18*, *CYP4H17*, *CYP4C36*). However, expression levels of key genes vary geographically such as the P450 *CYP6M7* [Fold-change (FC) = 115.8 (Arua) vs 24.05 (Tororo) and 16.9 (Kisumu)]. In addition, several genes from other families were also over-expressed including Glutathione S-transferases (GSTs), carboxylesterases, trypsin, glycogenin, and nucleotide binding protein which probably contribute to insecticide resistance across Uganda and Kenya. Genotyping of 17 microsatellite loci in the five locations provided evidence that a geographical shift in the resistance mechanisms could be associated with patterns of population structure throughout East Africa. Genetic and population structure analyses indicated significant genetic differentiation between Arua and other localities (F_ST_>0.03) and revealed a barrier to gene flow between Arua and other areas, possibly associated with Rift Valley.

**Conclusion:**

The correlation between patterns of genetic structure and variation in gene expression could be used to inform future interventions especially as new insecticides are gradually introduced.

## Background

Malaria remains one of the main causes of morbidity and mortality in Sub-Saharan Africa, predominantly in children under 5 years and pregnant mothers [1]. The transmission of these malaria-causing parasites to human is caused by *Anopheles* mosquitoes of which four species (*An*. *gambiae*, *An*. *coluzzii*, *An*. *funestus*, *An*. *arabiensis*,) have been identified as the major malaria vectors in Africa. In Uganda, the main malaria vectors are *Anopheles funestus*, *An*. *gambiae* sensu strict and *An*. *arabiensis* [2].

Malaria control in Uganda relies heavily on vector control using long-lasting insecticide nets (LLINs) and indoor residual spraying (IRS) mostly in regions of seasonal transmission [1]. The success of such interventions requires a good knowledge of vector populations particularly their susceptibility status to the main insecticides used for such control programs. The two major causes of insecticide resistance are alterations in the target sites and an increase in the rate of insecticide metabolism [3–5]. In Uganda, the previous investigation of pyrethroid resistance has revealed the absence of knockdown resistance (*kdr*) target-site mutation in *An*. *funestus* [4, 6, 7]. The underlining molecular basis of resistance to the insecticide for this vector in Uganda has been associated with cytochrome P450 over-expression in the eastern part (Tororo) [3] but it remains to establish if the same mechanisms are present country-wide and in neighboring Kenya [3]. It is important to know whether the resistance front is unique, or gene flow is uniform across the region. Previous efforts to characterize the mechanisms of pyrethroid resistance in *An*. *funestus* has revealed that resistance is mainly driven by metabolic resistance [8–10]. Cytochrome P450s are known to be a primary enzyme family conferring resistance to pyrethroids in malaria vectors [11]. Molecular studies conducted in Malawi and Mozambique have revealed that the duplicated P450 genes, *CYP6P9a*, and *CYP6P9b* are the main genes driving pyrethroid resistance in this region [10, 12–15]. In addition, the studies performed in Malawi have revealed a similarly minor role of *CYP6P9a* and *CYP6P9b* [7]. Recently, molecular studies conducted on *An*. *funestus* in southern Africa (Malawi), East (Uganda), and West Africa (Benin) have revealed that the duplicated P450 genes (*CYP6P9a* and *CYP6P9b*), which were highly overexpressed in southern Africa, were not the most upregulated in other regions, where other genes, including GSTe2 in West (Benin) and *CYP9K1* in East (Uganda) [3], were overexpressed. This variation of expression profiles in Africa suggests that the molecular basis of pyrethroid resistance might vary across Africa and within national populations of *An*. *funestus*. However, the molecular basis of pyrethroid metabolic resistance in *An*. *funestus* across Uganda remains poorly characterized [4, 6].

It also remains unknown whether the same control strategy could efficiently control *An*. *funestus* populations throughout Uganda and the neighboring countries. This is further the case in the context of ecological landscape changes such as the Rift Valley that spans East Africa and previously shown to restrict gene flow in *An*. *gambiae* [16, 17]. Indeed, based on microsatellite markers, the magnitude of genetic differentiation (*F*_ST_) between populations on opposite sides of the continent (~6000 km apart) was ~0.03, while the corresponding value between populations on either side of the Rift Valley (~400–500 km) was ~0.1 [17–20]. The genetic structure of *An*. *funestus* across Uganda remains poorly characterized in the context of the spread of insecticide resistance and impact of the Rift valley although a recent study highlighted a homogeneity between populations from Central and North Uganda [21] Assessing how mechanisms of pyrethroid resistance vary countrywide and whether it is linked to the genetic structure vector populations is an important step in designing nationwide vector control strategies. Furthermore, screening more populations could detect new genes driving such pyrethroid resistance. Identifying the full set of genes involved in resistance will help decipher the molecular basis of resistance and potentially identify resistance markers which can be used in the design of DNA-based molecular diagnostic tools for quick detection and tracking resistance in the field as recently done for P450-mediated metabolic resistance in southern African populations of *An*. *funestus* [12, 13].

In this present study, using microarray genome-wide transcription analysis, we characterized the molecular basis of pyrethroid resistance in *An*. *funestus* in Uganda and Kenya. We also provide evidence, using 17 microsatellite markers, that the genetic structure of the *An*. *funestus* in both countries varied and correlates with changes in gene expression.

## Materials and methods

### Study sites and samples

*An*. *funestus* mosquitoes were collected between 06.00 AM and 12.00 PM, from four districts in Uganda; Arua (Ar) in North West (3°1′N, 30°54′E), Bulambuli (Bl) in North-East (1°10′N, 34°23′E), Lira (Lr) in North Central (2°14′N, 32°54′E), and Tororo (Tr) in East (0°41′N, 34°10′E). A similar collection was made from Kisumu-Siaya district (0°05′S, 34°15′E) in West Kenya. Mosquito collections were carried out between December 2011 and June 2012: between the end of dry season and beginning of the rainy season, with temperatures and relative humidity ranging from 26°C to 29°C and 66% to 77% respectively. Indoor resting females were collected randomly in different locations using electric aspirators as described previously [4]. No specific permissions were required for these locations/activities and these field collections did not involve endangered or protected species. The blood-fed F_0_ adults were morphologically identified as belonging to the *An*. *funestus* group according to the key of Gilles and Coetzee (1987) [22]. They were left to become gravid and forced to lay eggs using the forced-egg laying method (Morgan et al 2010). A PCR assay was performed using the protocol of Koekemoer [23] to confirm that collected F_0_ adults were *An*. *funestus s*.*s*. [4]

### Resistance profile of different populations districts

The resistance patterns of the five populations districts in Uganda and Kenya to 0.75% permethrin insecticides was determined as described previously by Mulamba et al., [4] following the World Health Organization (WHO) protocol [24]. The Arua, Tororo, and Kisumu population of *An*. *funestus* are highly resistant to permethrin (27% mortality, 33% mortality, and 20% mortality, respectively for Arua, Tororo, and Kisumu after 1 hr exposure) [4]. The Bulambuli and Lira population of *An*. *funestus* are also resistant to permethrin (49% mortality and 51% mortality respectively for Bulambuli and Lira, after 1hr exposure) and the final mortality was assessed after 24 h [4].

### Detection of pyrethroid resistance genes using microarrays and qRT-PCR

Genes associated with pyrethroid resistance in the five locations were detected using the 8 X 60K Agilent microarray chip customarily designed for *An*. *funestus* as previously described [10]. This chip designed through the Array program (Agilent) (A-MEXP-2374) and previously described by Riveron et al [10], is made of sets of 15,527 transcripts generated from de novo transcriptome analysis [25], 8,540 Expressed Sequence Tags (ESTs) [26]. It also includes a set of 2850 *An*. *funestus* cDNAs from GenBank, a set of P450 genes from the *rp1* and *rp2* QTL BAC sequence [14, 27]; and 13,000 transcripts of the complete *An*. *gambiae* genome. Total RNA was extracted from three pools of 10 female mosquitoes per phenotype notably in Lira for which mosquitoes included: Control (unexposed to insecticide, C), Resistant [alive R) after exposure to 0.75% permethrin], and Susceptible (FANG susceptible colony; S). In other locations, the resistant (R) sample was included. The extraction was performed using the Picopure RNA isolation Kit (Arcturus, Applied Biosystems, Carlsbad, CA, USA). The RNA extraction was performed as previously described by Riveron et al., 2017 [3]. The quantity and quality of extracted RNA were assessed using a NanoDrop ND1000 spectrophotometer (Thermo Fisher Scientific, Waltham, MA, USA) and a Bioanalyzer (Agilent, Santa Clara, CA, USA), respectively. The Agilent Quick Amp Labeling Kit (two-color) was used to amplify the complementary RNA (cRNA) from each sample according to the manufacturer’s protocol and as described previously [3]. Resistant cRNAs (R) were labeled with cy5 dye, whereas susceptible cRNAs from the strain FANG (S) were labeled with the cy3 dye. The quantity and quality of all cRNAs were assessed using the NanoDrop and Bioanalyzer before labeling. Labeled cRNAs were hybridized to respective arrays for 17 h at 65°C following the manufacturer’s protocol. For each location and comparison, five hybridizations were performed by swapping the biological replicates.

Agilent GeneSpring GX 13.0 software was used to analyze the microarray data. The differentially expressed genes were identified using a threshold of twofold-change (FC) and a statistical significance of P<0.05 with Benjamin-Hochberg correction for multiple testing. The microarray data from this study is deposted in the Array Express under accession no. E-MTAB-9529. Finally, quantitative reverse transcription PCR (qRT-PCR) assays were performed to validate microarray results for the key candidate genes; 1 μg of RNA from key resistance genes, comparing the permethrin-resistant mosquitoes to FANG susceptible (R-S) mosquitoes, was used as a template for complementary (cDNA) synthesis using the superscript III (Invitrogen, Carlsbad, CA, USA) following the manufacturer’s guide. The qRT-PCR was carried out as previously described [10, 28] with the relative expression level and fold change of each target gene calculated according to the 2^−ΔΔCT^ method after normalization with the housekeeping genes, the ribosomal protein S7 (*RSP7*; AFUN007153) and actin5C (AFUN006819) [29].

### Genetic population structure of Anopheles funestus in Uganda and Kenya

Randomly field-collected F_0_ females confirmed as *An*. *funestus s*.*s* were genotypes at seventeen microsatellites loci genome-widely distributed [30, 31].

The mosquitoes from Uganda and Kenya samples (N = 43 in Arua, N = 48 in Bulambuli, N = 43 in Lira, N = 47 in Tororo, N = 26 in Kisumu) were genotyped as previously described by [7]. Briefly, genomic DNA extracted from F0 mosquitoes were used to amplify the 17 microsatellite loci (both di- and tri-nucleotide repeats) using 1.5 μl of reaction Buffer, 0.2 μl of dNTP mix (25 mmol), 0.325 μl of both the forward (included a 19bp tag) and reverse primers, 0.2 μl of Hot Start Taq (Qiagen Inc.), 1 μl of MgCl_2_ and 1μl of genomic DNA (15ng/ul). S1 Table contains the list of loci and their primers. PCR conditions were: 5min at 95°C followed by 35 cycles of denaturation at 94°C for the 30s, annealing at 58°C for 30s, and extension at 72°C for 30s, with a final extension step at 72°C for 10min. The Beckman Coulter CEQ8000 was used for the fragmented sizing. Micro-Checker version 2.2.3 [32] was used to check for the null allele and scoring errors.

For each locus and each population sample, heterozygosity was computed using GENETIX v.4.05 [33] and the number of alleles was computed using FSTAT v.2.9.3.2 [34]. FSTAT and GENEPOP v.4.0.3 [35] were used to assess the deviation from Hardy-Weinberg equilibrium at each locus, each population sample, and overall as indicated by the inbreeding coefficient (*F*_*IS*_). Linkage disequilibrium between pairs of microsatellite loci was assessed using FSTAT v.2.9.3.2 [34]. Significance was tested using the randomization approach implemented in FSTAT with Bonferroni-adjusted *P-*values. Genetic differentiation between populations was assessed by estimating Wright's F-statistics [36], calculated according to Weir & Cockerham [37]. Statistical significance of *F*_*ST*_ was assessed using G-based exact tests for genotypic differentiation [34], available in GENEPOP.

We applied a Bayesian model-based clustering algorithm to infer population structure and to assign individuals (probabilistically) to clusters without a priori knowledge of population units and limits. This approach, implemented in STRUCTURE v 2.3.4 [38], uses individual multilocus genotype data to cluster individuals into K groups while minimizing the Hardy-Weinberg disequilibrium and gametic phase disequilibrium between loci within groups [39]. In STRUCTURE v 2.3.4, the number of distinct genetic clusters in the data set (K) was estimated from 1 to 5 by the posterior log probability of data under each K, Ln [Pr (X|K)] [38]. Each run carried out 100,000 iterations after a burn-in period of 100,000, using the admixture model and correlated allele frequencies. To check for convergence of the Markov chain Monte Carlo (MCMC), we performed over 5 replicates for each value of K and then checked the consistency of results [40]. The method of Evanno et al., was used to determine the most likely number of clusters [41].

The correlation between genetic and geographical distances was assessed by the regression of *F*_*ST*_ / (1-*F*_*ST*_) on the logarithm (ln) of geographical distance [42], and tested using the Mantel test available in GENEPOP.

Kruskal-Wallis test was used to compare the average number of alleles and the average proportions of heterozygosity between populations using GraphPad Prism 5. The Bonferroni correction procedure [43] was applied to evaluate significance when multiple tests were performed.

## Results

### Transcription profiling of the pyrethroid resistant population of Lira

**Fig 1:**
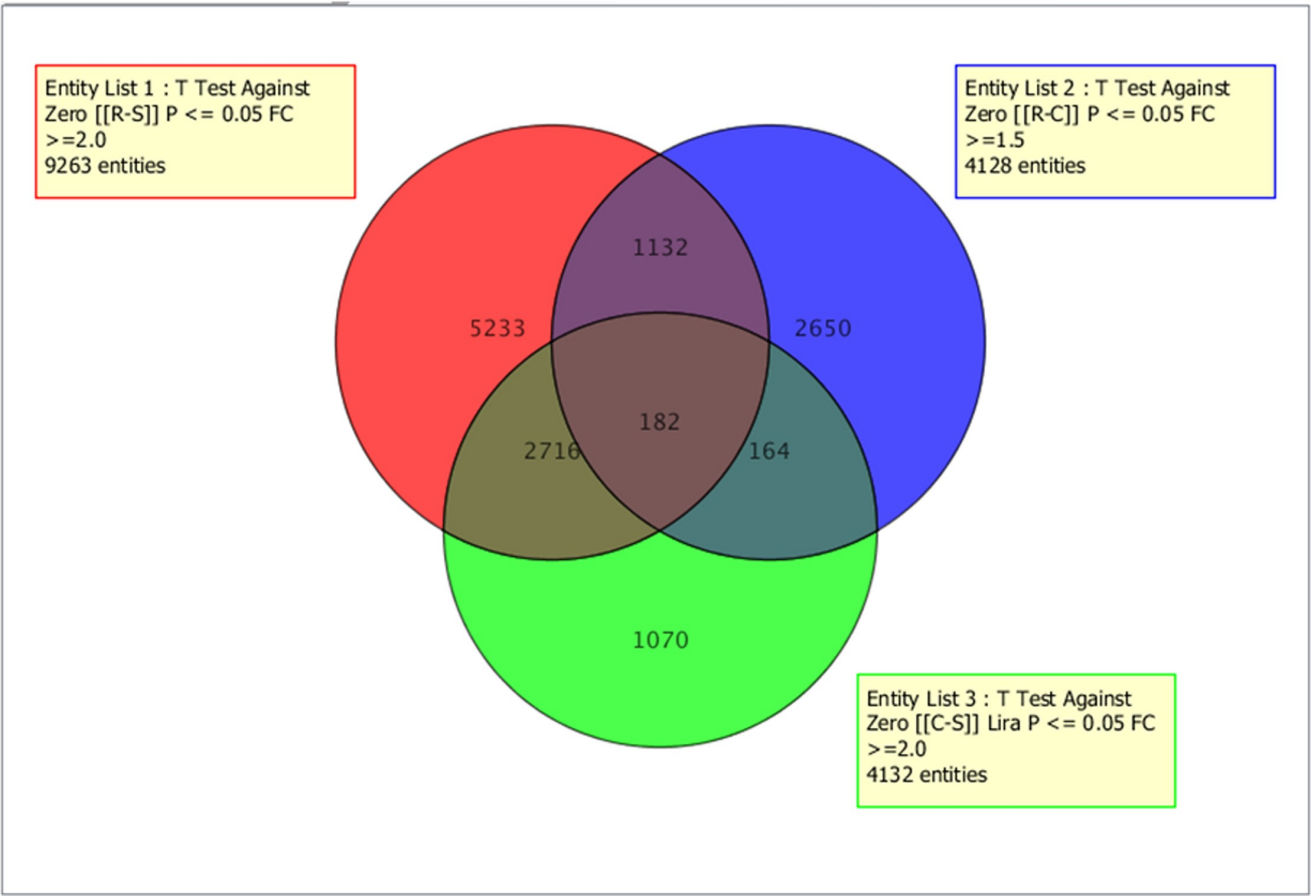
Venn-diagram summarizing the number of probes differentially expressed in each and between comparisons in Lira at fold-change (FC)>2 and p<0.05 in R-C, R-S, and C-S comparisons, as well as the commonly expressed probes.

The triangular hybridization performed in Lira between mosquitoes resistant to permethrin (R), unexposed to insecticide (C), and the FANG susceptible laboratory strain (S) allows a comparative analysis of transcription profile in this location. A high number of the probes were significantly differentially expressed (p<0.05) for the R-S (9263), R-C (4132 with fold-change of 1.5), and the C-S (4128) (Fig 1). However, a Venn-diagram analysis revealed that only 182 probes were commonly differentially expressed in all three groups (Fig 1).

#### Genes commonly overexpressed in R-C, C-S, and R-S strains

Among these, the probes with the highest over-expression in the R-S comparison (FC79.8) and other included a hypothetical protein ortholog of the AGAP000603 in *An*. *gambiae* with an unknown function. The nucleotide-binding protein 2 (nbp2) (Afun008887) gene was also consistently overexpressed in all three comparisons although with the highest fold change seen in R-S (FC: 17.15) (Table 1). This gene was also significantly overexpressed in R-C and C-S with a much lower FC value (FC = 1.7; FC = 7.2 respectively for R-C and C-S) (Table 1). The sg2 salivary protein (EE589890.1) corresponding to AFUN016226 was also commonly over-expressed. The sulfotransferase gene (Afun013871) and the aldehyde oxidase (AGAP006225) were other detoxification genes commonly expressed in R-C, R-S, and C-S with a similar fold change (Table 1).

**Table 1:** List of top detoxification genes significantly overexpressed in pyrethroid-resistant *An*. *funestus* in Lira for all comparisons.

Systematic Name	Gene Name	R-C	R-S	C-S	Description
Afun014076	NA	4.4	79.8	16.05	Hypothetical protein (AGAP000603)
Afun008887	NA	1.74	17.15	7.22	Nucleotide-binding protein 2 (nbp 2)
EE589890.1	AFUN016226	1.5	6.1	3.01	Sg2 salivary protein
Afun000408	NA	1.96	5.7	3.7	Heat shock cognate 70 protein
gb-Aldehyde_oxidase	AGAP006225	1.9	2.3	3.3	Aldehyde oxidase
Afun013871	NA	2.2	2.2	2.9	Sulfotransferase
Afun009492	NA	2.85	15.16		Carboxylesterase
AGAP002418-RA	CYP4D15	3.17	2.29		Cytochrome p450
gb-CYP4H19	CYP4H19	2.16	2.48		Cytochrome p450
CYP6M1b.fixed.seq	NA	1.86	2.52		Cytochrome p450
Afun008909	CYP4K2	1.58	2.11		Cytochrome p450
Afun004223	CYP4H17	4.08	7.45		Cytochrome p450 4d1
Afun001392	NA	3.68	15.29		Glycine dehydrogenase
gb-CYP9K1	CYP9K1		13.68	31.80	Cytochrome p450
gb-CYP9J3	CYP9J3		5.44	4.38	Cytochrome p450
Afun012197	CYP304B1		4.31	4.62	Cytochrome p450
Combined_c6791	CYP9J11		3.72	2.72	Cytochrome p450
Afun006930	CYP6M7		3.91	2.93	Cytochrome p450 6a8
Afun007769	CYP9K1		11.00	14.49	Cytochrome p450 cyp9k1
Afun000143	CYP9K1		2.29	6.68	Cytochrome p450 cyp9k1
Afun008354	GSTD3		3.80	3.57	Glutathione transferase (agap004382-pa)
Afun008293	NA		133.61	86.53	Trypsin-related protease
Afun015032	CYP302A1	2.72			Cytochrome p450
gb-CYP6M2	CYP6M2	2.58			Cytochrome p450
gb-CYP4C25	CYP4C25	2.33			Cytochrome p450
Afun005448	CYP302A1	2.26			Cytochrome p450
AGAP000194-RA_Cytoch. . .	CYP4C25	2.23			Cytochrome p450
gb-CYP4C25	CYP4C25	2.09			Cytochrome p450
Afun007450	GSTE2	2.62			Glutathione s-transferase
Afun007478	GSTE3	2.79			Glutathione-s-transferase gst
AGAP009193-RA_Glutat. . .	GSTE4	2.10			Glutathione-s-transferase gst
Combined_c920	GSTE2	1.87			Glutathione-s-transferase gst
Combined_c920	GSTE2	1.68			Glutathione-s-transferase gst
Afun009227	NA		36.92		Argininosuccinate lyase
Afun013921	NA		33.04		Chymotrypsin 1
Combined_c1486	CYP6AH1		8.65		Cytochrome p450
CYP6M4.seq	CYP6M4		5.25		Cytochrome p450
Combined_c1486	CYP6AH1		5.19		Cytochrome p450
Afun010630	CYP6P5		4.72		Cytochrome p450
Combined_c6791	CYP9J11		4.43		Cytochrome p450
gb-CYP4H16	CYP4H16		4.21		Cytochrome p450
Afun007127	CYP4C36		4.07		Cytochrome p450
gb-CYP6M3	CYP6M3		2.59		Cytochrome p450
Afun004392	CYP6M3		2.47		Cytochrome p450
Afun009522	CYP9J11		2.17		Cytochrome p450
Afun007663	CYP6M3		51.54		Cytochrome p450 6a8
Afun000798	CYP6M3		2.92		Cytochrome p450 6a8
Afun009584	CYP6M3		2.21		Cytochrome p450 6a8
CD577407.1	GSTE2		3.56		Glutathione s-transferase
Afun007499	GSTD1		2.30		Glutathione transferase
Afun013481	GSTE1		4.44		Glutathione-s-transferase gst
Afun009866	GSTE6		3.04		Glutathione-s-transferase gst
Afun011042	NA		8.53		Glycine dehydrogenase
gb-COEAE2A	COEAE2A			2.48	Carboxylesterase
Afun007469	CYP9J4			3.95	Cytochrome p450
Afun012666	CYP314A1			3.34	Cytochrome p450
Afun007369	CYP6P9b			3.25	Cytochrome p450

#### Genes commonly overexpressed in R-S and C-S strains

Several detoxification genes or resistance-related genes were commonly and significantly overexpressed in R-S and C-S strains. Among the most overexpressed genes commonly observed in R-S and C-S were proteases such as a trypsin-related protease (Afun008293), which was the top upregulated with FC 133.61 in R-S and 86.53 in C-S. Several detoxification genes were commonly upregulated in both strains, with cytochrome P450s being the most; notably, CYP9K1 (three probes) overexpressed with FC 13.68 in R-S and 31.80 in C-S. Two other genes CYP9J11 and CYP9J3 were also up-regulated with FC 5.44 in R-S and 4.38 in C-S for CYP9J11 and FC 3.72 in R-S and 2.72 in C-S for CYP9J3. CYP6M7 was also overexpressed with FC 3.90 in R-S and 2.94 in C-S (Table 1). Glutathione-S-transferases (GSTs) were also significantly overexpressed in pyrethroid-resistant mosquitoes from Lira compared to the susceptible FANG strain, notably GSTe3 (Afun008354) (FC 3.8 and 3.6, in R-S and C-S respectively) (Table 1).

#### Genes commonly overexpressed in R-C and R-S strains

A set of five transcripts belonging to cytochrome P450 genes were commonly upregulated in R-S and R-C, with CYP4H17 (FC 7.44; 4.07), CYP4H19 (FC 2.47; 2.16), CYP4K2 (FC 2.1; 1.57), CYP4D15 (FC 2.28; 3.17), and CYP6M1b (FC 2.5; 1.8) (Table 1). The transcript Afun009492 and Afun001392 belonging to carboxylesterase and glycine dehydrogenase genes respectively were also overexpressed in both R-S (FC 15.16; 15.28 respectively) and C-S (FC 2.84; 3.6 respectively).

Probes from other detoxification genes were uniquely upregulated in a comparison. Those found only in R-S included the cytochrome P450s: CYP6M7 (five probes), *CYP6AH1* (two probes), *CYP6M4*, *CYP6P5*, *CYP9J11* (two probes), CYP4H16, and CYP4C36. Three other genes, argininosuccinate lyase (FC, 36.9), chymotrypsin 1 (FC, 33.03), glycine dehydrogenase (FC, 8.53) (Table 1) and four Glutathione-S-transferases (GSTs) GSTe1 GSTe2, GSTe6, and GSTD1, were upregulated only in the R-S strain. Genes only present in the C-S strain included carboxylesterase (COEAE2A, FC 2.48), CYP9J4 (FC 3.94), CYP314A1 (FC 3.34) and CYP6P9b (FC 3.25) (Table 1). Other detoxification genes, including the cytochrome P450s *CYP302A1* (two probes) *CYP6M2*, *CYP4C25* (three probes), and three glutathione-S-transferases (GSTs) GSTe2 GSTe3, GSTe4, were upregulated only in the R-C strain (Table 1).

### Comparison of expression profiles between Arua, Bulambuli, Lira, Tororo in Uganda, and Kisumu in Kenya

**Fig 2:**
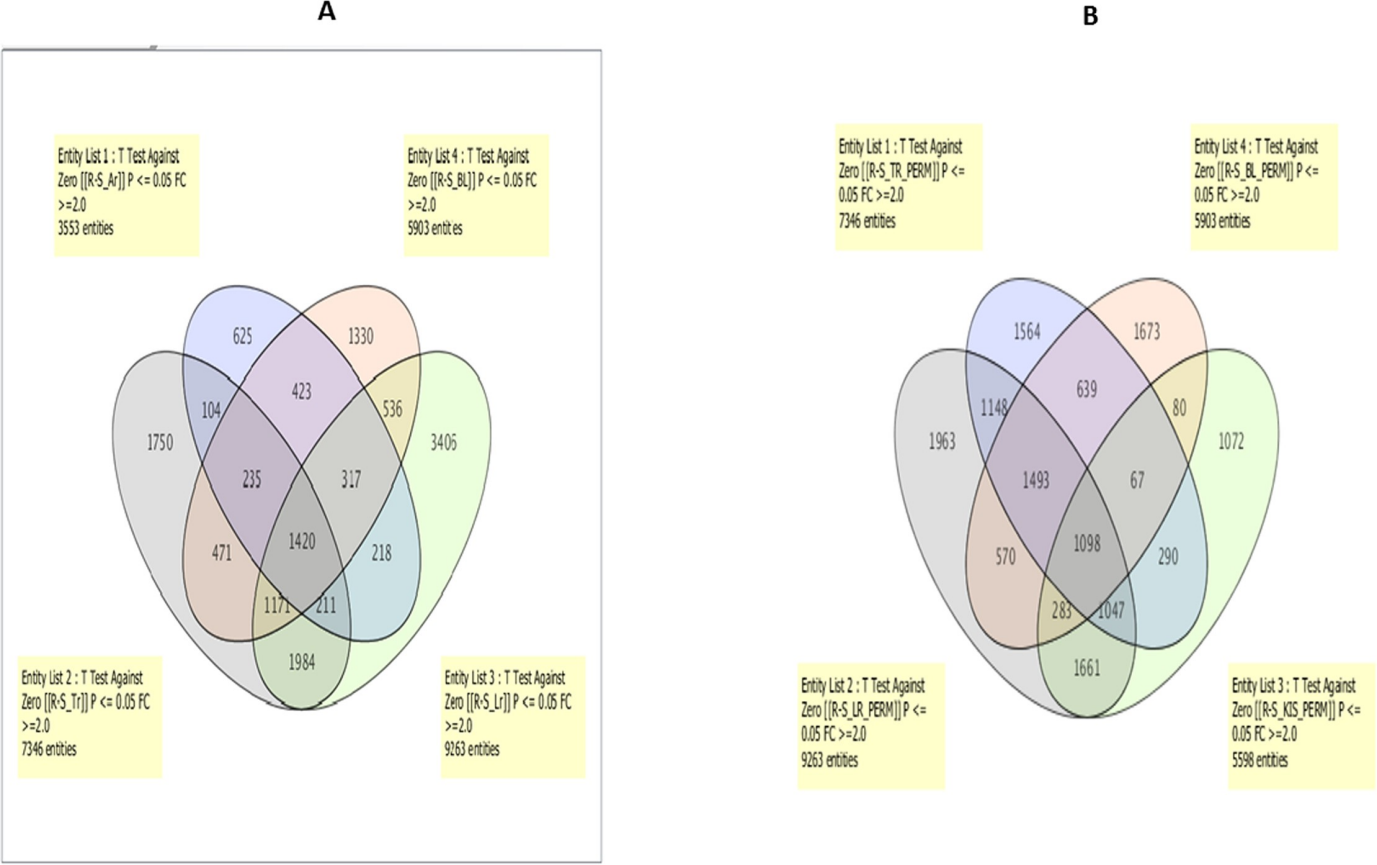
Transcription profile of pyrethroid resistance. **A.** Venn-diagram of the comparison between Arua, Bulambuli, Lira, and Tororo for the R-S comparison only. **B.** Venn-diagram of the comparison between Bulambuli, Lira, Tororo, and Kisumu for the R-S comparison only.

To detect the set of genes associated with permethrin resistance across Uganda and neighboring Kenya, the same 8X60k microarray chip was used to compare mosquitoes alive after permethrin exposure and compared to the full susceptible laboratory strain (R-S). The number of probes that were differentially expressed (>2-fold change, FC) between R and S mosquitoes for each locality and between them is indicated in Fig 2 (*P* < 0.01). Overall, 3553 probes were differentially expressed in the Arua population, 5903 in the Bulambuli population, 9263 in the Lira population, 7346 in the Tororo population and 5598 in Kisumu. When comparing the four Uganda populations, a total of 1420 probes were commonly differentially expressed (Fig 2A) whereas 1098 probes were commonly differential expressed when Kisumu was compared to Lira, Tororo and Bulambuli (Fig 2B).

#### Genes commonly up-regulated in the five locations (Arua, Bulambuli, Lira, Tororo, and Kisumu)

Among the detoxification genes, several cytochromes P450s were most commonly overexpressed in all the five locations. Most of these P450 genes showed a similar level of expression in all the five localities and included *CYP6Z1* (two probes), *CYP9K1*, and *CYP6AG1*. However, the overexpression levels of some candidate genes show significant geographical variation between locations. In Arua, *CYP6M7* (FC 115.88) has the highest overexpression levels than other locations. Another P450, *CYP4H18*, although commonly expressed in all five localities, was significantly present in Arua than other localities, suggesting a bigger role for this gene in Arua and supporting a differential expression in Arua compared to other Uganda locations.

This shift in expression pattern was also observed for other genes commonly overexpressed in all five locations. These include Afun000500 (glycogen) and Afun007894 (Trypsin) with the highest overexpression in Arua (FC 39.71 and FC 4.96) (Table 2).

**Table 2:** Detoxification genes commonly upregulated in Arua, Bulambuli, Lira, Tororo and Kisumu localities in Uganda and Kenya.

Systematic Name	Gene Name	AR	BL	LR	TR	KS	Description
Afun008347	NA	4.96	3.35	2.69	3.88	3.30	Chymotrypsin 1
Afun015244	Cuticular protein	13.43	15.97	7.81	5.91	5.85	Cuticular protein
Afun007769	CYP9K1	15.19	16.28	11.00	16.12	17.63	Cytochrome p450
CYP6Z1	CYP6Z1	4.97	3.65	2.65	3.11	4.81	Cytochrome p450
CYP6Z1	CYP6Z1	4.08	3.00	2.05	2.53	3.65	Cytochrome p450
Afun009335	CYP6AG1	3.02	2.88	2.27	2.77	3.64	Cytochrome p450
Afun006930	CYP6M3	2.29	4.39	3.91	5.26	4.20	Cytochrome p450
Afun007663 (CYP6M7)	CYP6M7	115.88	108.96	51.54	24.05	16.98	Cytochrome p450
Afun012343	CYP4H18	7.39	6.84	4.23	4.35	4.14	Cytochrome p450
Afun013481	GSTE1	7.45	4.22	4.44	5.61	8.87	Glutathione-s-transferase GST
Afun000500	NA	39.71	31.58	16.74	23.02	17.29	Glycogenin
Afun008887	Cytosolic Fe-S cluster assembly	16.22	14.56	17.15	17.64	15.00	Nucleotide binding Protein 2 (nbp 2)
Afun007894	NA	5.00	3.09	3.20	4.10	3.46	Trypsin delta gamma
Alpha_Carboxylase	Carboxylesterase	3.29	3.31	2.05	3.07		Carboxylesterase
Afun007575	NA	2.55	2.88	3.83	3.66		Chymotrypsin-like protein
Afun004223	CYP4H17	21.68	11.55	7.45	7.51		Cytochrome p450
Afun007549	CYP9K1	15.10	12.62	13.68	6.64		Cytochrome p450
Afun007127	CYP4C36	10.55	4.44	4.07	2.64		Cytochrome p450
Combined_c6791	CYP9J11	5.66	6.43	4.43	4.19		Cytochrome p450
Afun000143	CYP9K1	5.90	4.74	2.29	2.38		Cytochrome p450
Afun013871	NA	5.39	3.37	2.21	2.29		Sulfotransferase
Combined_c3002	CuSOD3	3.13	2.78	2.28	2.48		Superoxide dismutase
Combined_c3002	CuSOD3	3.11	2.82	2.27	2.44		Superoxide dismutase
Afun008293	NA	49.63	115.16	133.61	83.03		Trypsin-related protease
CYP6Y2_rvcpl.seq	CYP6Y2	3.18	3.23	2.33		3.68	Cytochrome p450
CYP6Y2_rvcpl.seq	CYP6Y2	3.12	2.86	2.53		3.50	Cytochrome p450
Afun015895	CYP4H25	9.22	3.04		5.34	2.74	Cytochrome p450
Afun013921	NA		11.79	33.04	49.49	22.30	Chymotrypsin 1
CYP6M4.seq	CYP6M4		3.73	5.25	9.17	4.99	Cytochrome p450
Afun012197	CYP304B1		3.03	4.31	3.29	2.90	Cytochrome p450
CYP6M1b.fixed.seq	CYP6M1		3.01	2.52	2.96	2.47	Cytochrome p450
Afun008909	CYP4K2		2.79	2.11	2.99	2.93	Cytochrome p450
gb-CYP4H16	CYP4H16		2.76	4.21	2.62	2.40	Cytochrome p450
gb-CYP9J3	CYP9J3		2.74	5.44	2.13	4.48	Cytochrome p450
Afun004392	CYP6M3		2.52	2.47	2.68	3.48	Cytochrome p450
Afun009584	CYP6M3		2.22	2.21	3.17	2.78	Cytochrome p450 6a8
Afun008354	GSTD3		5.56	3.80	5.07	11.06	Glutathione transferase (agap004382-pa)
gb-CYP325D1	CYP325D1	3.81	2.10				Cytochrome p450
CYP6P1	CYP6P1	3.47		2.87			Cytochrome p450
gb-CYP306A1	CYP306A1	4.33		4.47			Cytochrome p450 306a1
Afun003220	CYP6P9b	4.06		3.02			Cytochrome p450
Afun000045	GSTE2	3.06			2.15		Glutathione-s-transferase gst
Combined_c3045	NA	4.36			2.98		Glucose dehydrogenase
Afun012777	CYP4C36	19.17			10.33		Cytochrome p450
Afun015331	CYP307A1	14.12			3.94		Cytochrome p450 307a1
CYP6M3.seq	CYP6M3	5.75			3.11		Cytochrome p450
CYP6P9b	CYP6P9b	2.67				2.46	Cytochrome p450
CYP6z1	CYP6z1	2.01				2.87	Cytochrome p450
Combined_c1486	CYP6AH1		5.19	3.58			Cytochrome p450
AGAP005698-RA_Cuticular	CPTC 4		5.84		3.55		Cuticular
gb-CYP4J10	CYP4J10		3.21		2.74		Cytochrome p450
Afun009866	GSTE6		3.04		2.73		Glutathione-s-transferase gst
Combined_c1626	CYP9J3		2.72		2.34		Cytochrome p450
AGAP009375-RA_Cytoch. . .	CYP9M2		2.46		2.29		Cytochrome p450
Afun007499	GSTD1		2.30		2.06		Glutathione transferase
AGAP002418-RA_Cytoch. . .	CYP4D15		2.29		3.14		Cytochrome p450
Afun010909	CYP6AH1			2.01	2.90		Cytochrome p450
Alpha Carboxylase				3.20	2.90		Carboxylesterase
CD578169.1	NA			3.31	2.50		Trypsin
gb-COEAE6O	COEAE6O				2.07	2.02	Carboxylesterase
CYP6Z3rvcpl.seq	CYP6Z3				2.37	4.19	Cytochrome p450
Afun007678	CYP6Z4				2.35	3.26	Cytochrome p450
Afun011925	CYP4D22				2.12	3.38	Cytochrome p450
Afun010286	CYP4AR1				2.11	2.56	Cytochrome p450

**Footnote:** AR = Arua; BL = Bulambuli; LR = Lira; TR = Tororo; KS = Kisumu

The unique glutathione-s-transferase GST gene (GSTe1) commonly up-regulated in the five locations had high FC values in Arua and Kisumu (FC 7.45 and FC 8.87 respectively) (Table 2). Beside cytochrome P450s, other genes included a chymotrypsin 1 (Afun008347), cuticular protein or-1 family (Afun015244), nucleotide-binding protein 2 (nbp2) (Afun008887) and trypsin-related protease (Afun008293) were up-regulated (Table 2).

#### Genes commonly up-regulated only in the four Uganda locations (Arua, Bulambuli, Lira, and Tororo)

Among the commonly up-regulated detoxification genes, cytochrome P450s were the most predominant with five genes over-expressed; while only a single carboxylesterase was over-expressed. *CYP4H17* was the most over-expressed P450 gene with the highest FC value in Arua (FC 21.68) followed by Bulambuli, Tororo, and Lira with FC values of 11.55, 7.51 and 7.45, respectively. Another over-expressed P450 was CYP9K1 (Afun007549) with the highest expression level in Arua (FC, 15.10) followed by Lira, Bulambuli, and Tororo with FC values of 13.68, 12.62 and 6.64, respectively. *CYP4C36* was also overexpressed in Arua (FC 10.55) and had a similar expression level in Bulambuli and Lira (FC 4.44 and 4.07 respectively), whereas a lower fold change (FC 2.64) was observed in Tororo. *CYP9J11* (Ortholog of *CYP9J5* in *An*. *gambiae*) (Combined_c6791) had a similar expression level in the four localities (Table 2). The unique carboxylesterase gene commonly up-regulated in the four locations had comparatively similar low FC values ranging from 2.05 to 3.29 (Table 2). Beside cytochrome P450s, other over-expressed genes included: a chymotrypsin 1 (Afun008347), sulfotransferase (Afun013871), and trypsin-related protease (Afun008293) were also up-regulated (Table 2).

#### Genes commonly up-regulated only in all locations (Arua, Bulambuli, Lira, and Kisumu) but not in Tororo

Only *CYP6Y2* (two probes) gene was commonly over-expressed in four localities with a similar expression level (Table 2).

#### Genes commonly up-regulated in all locations (Arua, Bulambuli, Tororo, and Kisumu) but not in Lira

CYP4H25 was the most overexpressed gene with the highest FC value in Arua (FC, 9.22) followed by Tororo, Bulambuli, and Kisumu with FC values of 5.34, 3.04 and 2.74, respectively (Table 2).

#### Genes commonly up-regulated in all locations (Bulambuli, Lira, Tororo, Kisumu) but not Arua)

Among the commonly up-regulated detoxification genes, cytochrome P450s were the most predominant with eight genes over- expressed; while only a single glutathione-s-transferase GST gene (GSTD3) was over-expressed. Of the P450s, *CYP6M4* was the most overexpressed gene with the highest FC value in Tororo (FC 9.17) followed by Lira, Kisumu, and Bulambuli with FC values of 5.25, 4.99 and 3.73 respectively. Most of these P450 genes showed a similar level of expression in all the four localities and included *CYP304B1*, *CYP6M1*, *CYP4K2*, *CYP4H16*, *CYP9J3*, and *CYP6M3* (Table 2). The unique glutathione-s-transferase (GSTD3) gene commonly up-regulated in the four locations was overexpressed with the highest FC value in Kisumu (FC 11.06) followed by Bulambuli, Tororo, and Lira with FC values of 5.56, 5.07 and 3.80 respectively (Table 2).

#### Genes common only in two localities

Analysis of the list of genes commonly overexpressed in only two localities revealed that, for Arua and Bulambuli, only *CYP325D1* was observed and showed high expression in Arua. For those overexpressed only in Arua and Lira, the P450 *CYP6P1* and *CYP6P9b* were detected, although with higher expression in Arua (FC, 3.47 and 4.06 respectively) than in Lira (FC, 2.87 and 3.02 respectively). The *CYP306A1* was also upregulated and showed a similar level of expression in both localities. The list of genes overexpressed only in Arua and Tororo was dominated by the *CYP4C36* and *CYP307A1* with higher overexpression in Arua for both genes (*e*.*g*., FC 19.17 for *CYP4C36* in Arua *vs*. only FC 10.33 in Tororo and FC 14.12 for *CYP307A1* in Arua *vs*. only FC 3.94 in Tororo), suggesting that both genes are mainly driving resistance in Arua. The *GSTe2* was common to both localities with FC, 3.06 in Arua *vs*. FC, 2.15 in Tororo.

### Validation of the microarray data by qRT-PCR

**Fig 3:**
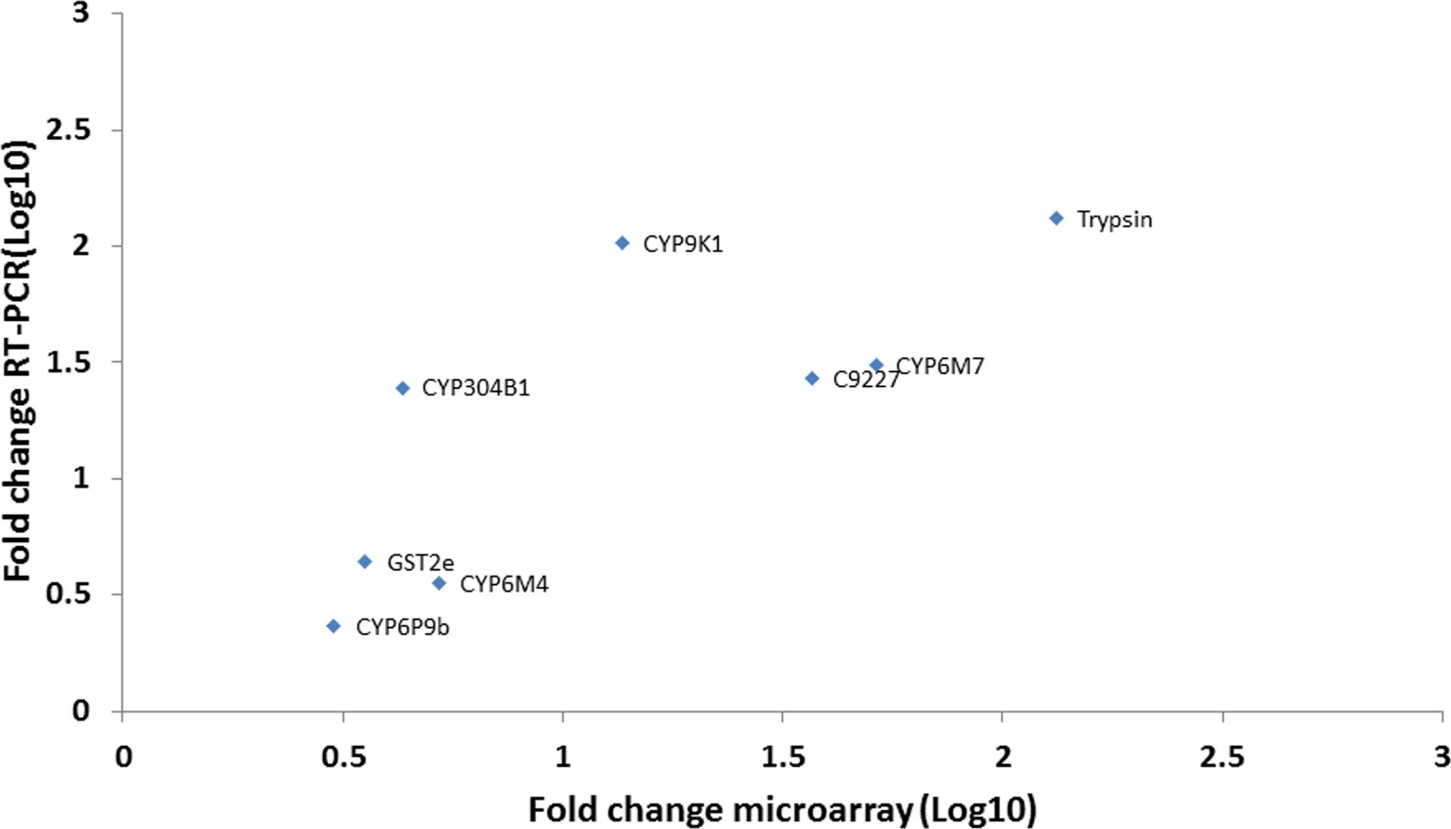
Correlation between microarray and qRT- PCR expression profiles.

Eight transcripts overexpressed in resistant samples including five cytochrome P450s (*CYP6M4*, *CYP9K1*, *CYP6P9b*, *CYP304b1*, *CYP6M7)*, one GST *(GSTe2)*, one protease (Trypsin1) and one carboxylesterase Afun009227 (c_9227) were selected for validation with microarray data using qRT-PCR. A positive and significant correlation (R^2^ = 0.8; p = 0.017) was recorded between qRT-PCR and microarray fold change measurements (Fig 3).

### Analysis of genetic population of Anopheles funestus in different locations

The significant differences in the gene expression profiles observed between populations of *An*. *funestus* in Uganda (notably between Arua and others) and Kenya could suggest the presence of barriers to gene flow that are affecting the spread of resistant genes. However, knowledge of the population genetic structure of *An*. *funestus* in Uganda and Kenya is limited.

#### Genetic diversity and Hardy-Weinberg equilibrium

Genotypes of *An*. *funestus* females were scored at 17 microsatellites loci. These microsatellite loci were highly polymorphic with a number of distinct alleles per locus ranging from 8 (AFUB12) to 20 (FUNO) for the combined five populations (Table 3). The average number of alleles per locus ranged from 7.1 (Kisumu) to 8.7 (Balambuli) and was not significantly different among populations (*P* = 0.92). However, Kisumu showed the lowest number of alleles for many loci with the minimum of 3 alleles observed for AFUB12 in this location. Mean observed heterozygosity across all loci ranged from 0.689 (Tororo) to 0.717 (Arua) and was not significantly different among populations (*P =* 0.85).

**Table 3:** Genetic diversity at 17 microsatellites loci in *Anopheles funestus* from Uganda and Kenya.

Locus				Populations			
			Uganda			Kenya	
		AR (N = 43)	BL (N = 48)	LR (N = 43)	TR (N = 47)	KS (N = 26)	All (N = 207)
AFND7	Nall	7	9	7	10	7	11
	He	0.729	0.756	0.777	0.74	0.775	0.775
	Fis	0.182	**0.184**	0.024	0.005	0.274	**0.125**
FUNR	Nall	9	8	4	6	7	13
	He	0.565	0.452	0.476	0.365	0.566	0.489
	Fis	**0.393**	**0.227**	**0.424**	0.136	**0.406**	**0.319**
FUNF	Nall	7	7	6	8	7	12
	He	0.65	0.665	0.689	0.735	0.708	0.704
	Fis	0.154	-0.085	-0.001	-0.06	-0.121	-0.001
AFND40	Nall	6	7	6	6	5	9
	He	0.747	0.754	0.745	0.722	0.698	0.744
	Fis	-0.015	-0.038	0.076	0.068	0.138	0.05
FUNO	Nall	9	9	7	10	6	20
	He	0.718	0.671	0.699	0.715	0.599	0.685
	Fis	**0.234**	0.142	0.181	-0.119	**-0.006**	**0.093**
AFUB10	Nall	11	10	9	10	9	15
	He	0.811	0.799	0.809	0.8	0.732	0.809
	Fis	**0.38**	**0.254**	**0.321**	0.16	0.283	**0.249**
FUNL	Nall	9	12	13	12	9	15
	He	0.834	0.799	0.848	0.777	0.8084	0.823
	Fis	**0.369**	**0.254**	**0.243**	0.134	0.21	**0.224**
AFND6	Nall	10	8	10	10	8	13
	He	0.825	0.787	0.836	0.802	0.805	0.828
	Fis	**0.251**	**0.164**	0.177	**0.11**	0.16	**0.148**
AFND32	Nall	10	9	12	10	10	13
	He	0.82	0.809	0.814	0.837	0.782	0.828
	Fis	0.077	0.186	-0.044	0.071	-0.013	**0.07**
AFND19	Nall	9	8	10	9	8	10
	He	0.835	0.774	0.755	0.702	0.751	0.767
	Fis	0.037	0.042	**0.211**	-0.05	0.2	**0.085**
FUNQ	Nall	7	7	7	6	7	11
	He	0.787	0.798	0.795	0.736	0.738	0.791
	Fis	0.214	**0.435**	**0.193**	**0.259**	**0.237**	**0.256**
AFND12	Nall	10	14	10	11	8	16
	He	0.848	0.848	0.856	0.832	0.828	0.847
	Fis	**0.243**	**0.249**	**0.224**	0.167	0.091	**0.196**
AFUB6	Nall	6	8	4	7	4	12
	He	0.325	0.315	0.288	0.302	0.522	0.364
	Fis	0.155	-0.113	0.045	-0.113	0.063	-0.006
AFUB11	Nall	6	7	11	7	4	12
	He	0.564	0.627	0.65	0.59	0.54	0.592
	Fis	-0.059	-0.152	-0.06	-0.035	0.166	-0.025
AFND30	Nall	11	12	11	11	12	14
	He	0.857	0.855	0.801	0.805	0.826	0.84
	Fis	0.036	**0.134**	-0.004	**0.296**	0.088	**0.1**
AFUB12	Nall	5	4	5	5	3	8
	He	0.634	0.601	0.629	0.566	0.401	0.586
	Fis	**0.314**	**0.385**	**0.345**	**0.221**	0.157	0.329
AFND5	Nall	8	9	7	7	7	11
	He	0.644	0.733	0.595	0.68	0.691	0.663
	Fis	-0.107	**0.073**	0.035	-0.083	0.074	**0.016**
**Mean**	**Nall**	8.235	8.705	8.176	8.529	7.1176	12.64
	**He**	0.717	0.708	0.71	0.689	0.6928	0.703
	**Fis**	0.171	0.149	0.141	0.077	0.141	**0.135**

All: refers to populations pooled. Nall, number of alleles. He expected heterozygosity under Hardy-Weinberg equilibrium. *F*_*IS*_ was calculated according to Weir & Cockerham. Bolded values: P < 0.05 after taking into account multiple tests.

AR = Arua; BL = Bulambuli; LR = Lira; TR = Tororo; KS = Kisumu

A significant heterozygosity deficit was observed in 29 out of 85 tests across the markers after Bonferroni correction at (P< 0.01) across the markers as shown by *F*_*IS*_ estimates (Table 3). Some markers such as AFUB12, FUNQ, and FUNR exhibited such heterozygosity deficit in 4 out of 5 locations suggesting that such deviation could be marker related. Kisumu had the lowest number of deficit (3/17) whereas Bulambuli had the highest (10/17) (Table 3).

When the pooled samples were analyzed as a single population, significant deviation from Hardy-Weinberg equilibrium (*F*_*IS*_ = 0.135; P<0.01) was observed within each population studied due to significant heterozygote deficiency (Table 3). No linkage disequilibrium was observed in any pair of loci after correction by the Bonferroni procedure (P>0.05) suggesting genetic independence between loci.

#### Genetic differentiation

**Fig 4:**
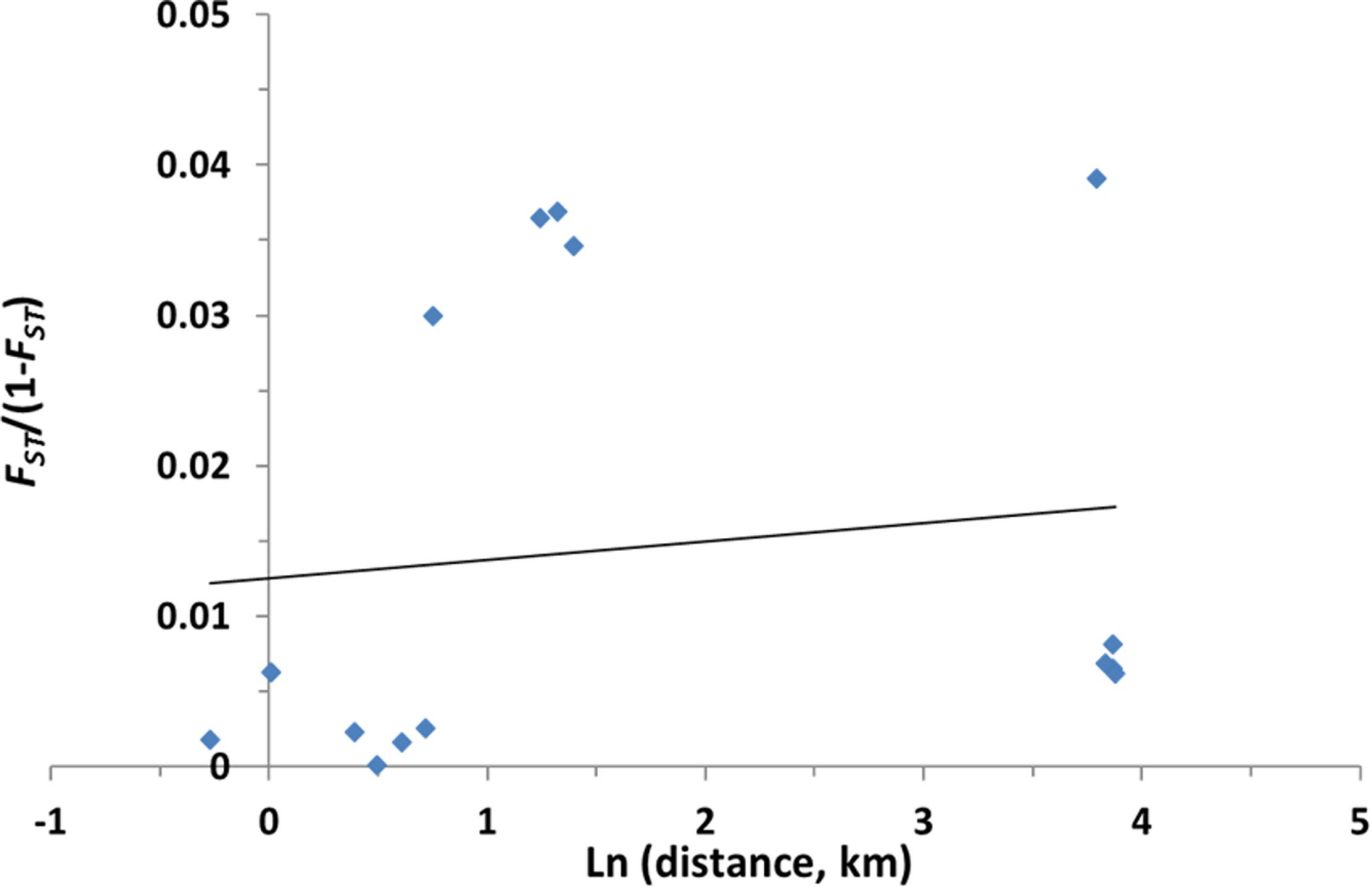
Correlation between average *F*_*ST*_ / (1-*F*_*ST*_) and the logarithm of geographical distance (in Km) for pairwise comparisons of 5 *Anopheles funestus* populations from Uganda and neighboring Kenya genotyped at 17 microsatellites loci.

**Fig 5:**
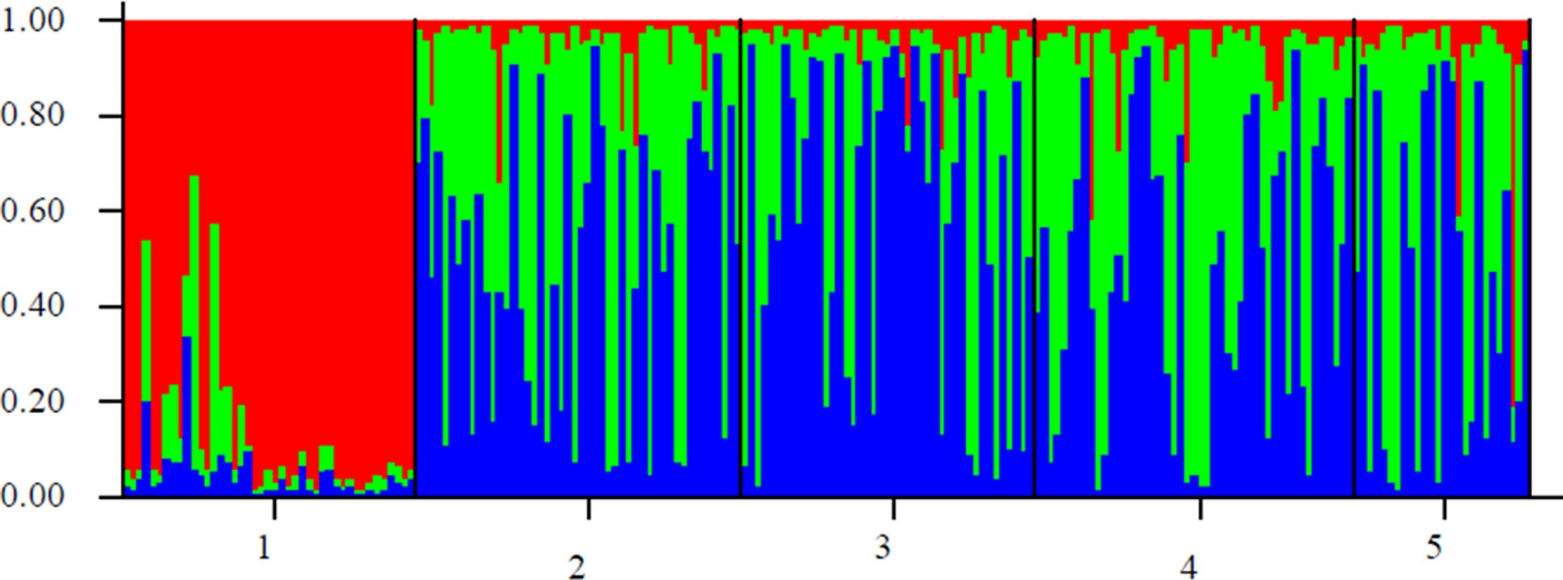
Bayesian cluster analysis using STRUCTURE: Graphical representation of the data set for the most likely k (k = 3), where each color corresponds to a suggested cluster and each individual is represented by a vertical bar. The numbers in the X-axis correspond to a specific sample: 1-Arua, 2-Bulambuli, 3-Lira, 4-Tororo, and 5 = Kisumu. The Y-axis represents the probability of assignment of an individual to each cluster.

The levels of genetic differentiation between pairs of populations were estimated by *F*_*ST*_ values. Table 4 shows *F*_*ST*_ estimates for all pairwise populations compared. The values of *F*_*ST*_ between pairwise population comparisons for all loci ranged from 0 (Bulambuli- Lira) to 0.037 (Arua- Kisumu). The highest significant *F*_*ST*_ estimates were obtained between Arua and other localities (Bulambuli, Lira, Tororo, and Kisumu) suggesting the presence of barriers to gene flow between these Arua and these locations (Bulambuli, Lira, Tororo, and Kisumu) (Table 4). Analysis of patterns of genetic differentiation at individual loci revealed higher *F*_*ST*_ estimates between Arua and other populations at some loci such as FUNO (0.27–0.32) and AFND6 (0.04–0.091). These two loci are located on the 2R chromosome at the vicinity of a major QTL (rp1; resistance to pyrethroid 1) previously associated with resistance to pyrethroids and close to a cluster of cytochromes P450s. This high *F*_*ST*_ estimates could suggest a difference in selection pressure between Arua and other locations in this genomic region in association with a difference in resistance mechanism supported by the variation in gene expression patterns from the microarray study. In contrast, the four locations of Bulambuli, Lira, Tororo, and Kisumu showed a very low and nonsignificant *F*_*ST*_ pairwise estimate, suggesting a high level of gene flow between these populations (Table 4). The segregation in the Arua population is similar to changes in gene expression profiles of resistance genes in this location. Furthermore, to understand the possible role of geographical distance in generating the genetic distance between Arua and other populations, the Mantel test was performed. The test revealed no significant correlation between the pairwise *F*_*ST*_ / (1-*F*_*ST*_) against the natural logarithm of pairwise geographical distance (R^2^ = 0.19, *P* = 0.806), suggesting that the population genetic structure of *An*. *funestus* in Uganda and Kenya did not conform to isolation by distance model (Fig 4) and suggesting that the difference in Arua may be caused by other factors. Finally, to confirm the estimated genetic differences inferred based on *F*_*ST*_, we performed Bayesian predictions of population structure. The population size was K = 3, predicted using the Evanno method (Fig 5). In agreement with the *F*_*ST*_ estimates, the Bulambuli, Lira, Tororo, and Kisumu samples from Uganda and Kenya shared a similar pattern whereas Arua forms a distinct cluster (Fig 5).

**Table 4:** Estimates of *F*_*st*_ values and their statistical significance.

	Arua	Bulambuli	Lira	Tororo
**Arua**				
**Bulambuli**	**0.0356**			
**Lira**	**0.0291**	0.0001		
**Tororo**	**0.0334**	**0.0018**	0.0025	
**Kisumu**	**0.0376**	**0.008**	0.0068	0.0062

*P-*values obtained after 10000 permutations; *P* < 0.001 for bolded values.

## Discussion

Insecticide resistance in *An*. *funestus* mosquitoes is spreading throughout Africa, threatening the success of malaria control methods. In this study, we characterized the mechanisms driving insecticide resistance in *An*. *funestus* population from Uganda and neighboring border town in Kenya. We further investigated the population genetic structure in the malaria vector *An*. *funestus* to help predict the pattern of spread of resistance and improve the design of insecticide resistance management strategies.

*An*. *funestus* populations in Uganda and neighboring Kenya are resistant to permethrin [4]. Microarray analysis identified several transcript coding for detoxification enzymes (P450s, GSTs, Carboxylesterase, Trypsin, Glycogenin, and nucleotide-binding protein) upregulated in the five localities. Among the commonly up-regulated detoxification genes, cytochrome P450s were the most predominant with several genes over-expressed as previously shown in several malaria vector populations [10, 27, 44, 45]. The predominant role of cytochrome P450 genes in the metabolic resistance observed in *An*. *funestus* population had been reported in several countries in Africa as the main detoxification enzymes implicated in pyrethroids resistance [46]. However, the difference in the expression levels of key P450s in Uganda and those reported on other Africa regions [3, 12] suggests that the origin of resistance is not the same across the countries, suggesting that independent selection events of resistance to pyrethroids have occurred in various populations [3]. A clear geographical difference was observed in the transcription profile of several detoxification genes between Arua and other locations in Uganda notable for the pyrethroid resistance gene, *CYP6M7*, previously shown to confer type I and II pyrethroid resistance in field populations of *An*. *funestus* in Africa [46, 47]. The role of this key metabolic gene showing geographical differences have also been observed in the same vector in Zambia [46], in a south-north transect in Malawi [47] and in Ghana [48], supporting that changes in resistance mechanisms between mosquito population countrywide.

Other P450 genes belonging to the CYP9 family were overexpressed in Arua including *CYP9K1* and *CYP9J11*, indicating a possible link to the increased pyrethroid resistance. *CYP9K1* had already been implicated in pyrethroid resistance in *Anopheles gambiae* in Africa [49]. In addition, few cytochrome P450s from the CYP6 family were also overexpressed (*CYP6P1*, *CYP6P9b*, *CYP6Y2*) although with low fold change but showing high expression in Arua. Surprisingly, one of the duplicated P450 genes *CYP6P9a*, which has been shown to play a main role in pyrethroid resistance in southern populations of *An*. *funestus* [10, 14] was not overexpressed in all the localities examined. The complete absence of overexpression of *CYP6P9a* indicates that the resistance mechanism in Uganda and neighboring Kenya is different from that observed in Malawi [3].

The reasons for the potential shift in the expression levels of the genes remain unknown but could be due to the nature of the selection that gave rise to the resistance. This shift in gene expression further highlights the genetic plasticity of natural populations of malaria vectors and their ability to adapt to various selection pressures. The finding of geographical differences in the role of key resistance genes in Arua suggests the presence of barriers to gene flow. *F*_*ST*_ values have shown a greater genetic differentiation between Arua and other localities (Bulambuli, Lira, Tororo, and Kisumu) suggesting the presence of barriers to gene flow. In addition, *An*. *funestus* populations from Uganda and neighboring Kenya are subdivided into two distinct genetic entities: Population around the Rift Valley (Arua) and populations from other side of the valley (Bulambuli, Lira, Tororo, and Kisumu). The existence of these two genetic entities was confirmed by different genetic approaches (i.e. Structure and genetic differentiation). This result confirms the fact that another factor explains the population genetic structure of *Anopheles funestus* in these localities. This observation suggests the impact of the Rift valley as a barrier to gene flow between populations of *An*. *funestus* in this region. Our results corroborate the previous microsatellite study in *An*. *gambiae* in Uganda, where Rift valley shows a great barrier to gene flow [16, 17]. However, the greater estimates of *F*_*ST*_ at two loci located at the vicinity of a known pyrethroid resistance genomic region (rp1) [14], suggests that the genetic differentiation observed between Arua and the other locations in Uganda could also be influenced by a difference in local selection as the FUNO and AFND6 loci have previously be shown to be under selection due to location of the cluster of pyrethroid resistance CYP6 P450 genes in this region [7, 47]. It will be useful in the future to investigate the selection sources of pyrethroid resistance in Arua, either from different agricultural practices or local vector control interventions, to establish if these can also account for the difference observed between this population and others.

The genetic test of isolation by distance suggested no significant correlation between genetic diversity and geographical distance confirming that this difference could be due to ecological or geographical factors [50]. The cause of this barrier could also be associated with the chromosomal differentiation because the difference of frequency for some inversions such as 3La was reported to impact the genetic structure of *An*. *funestus* [51].

When Arua was excluded, our results showed low levels of genetic differentiation between *An*. *funestus* populations (Bulambuli, Lira, Tororo, and Kisumu), suggesting that overall there is a high level of gene flow between populations of this species across most of Uganda showing a genes of interest such as insecticide resistance genes or future gene drive constructs, could spread quickly among populations of this vector. A previous study on the genetic structure of *An*. *funestus* populations using microsatellites markers implemented in other parts of Africa have already demonstrated the high gene flow [52–54]. The important gene flow between *An*. *funestus* populations from Bulambuli, Lira, Tororo, and Kisumu, revealed by our analysis, indicates the existence of inter-connected continuous populations of this malaria mosquitoes. Such observations were already reported in other genetic studies in the populations of *An*. *funestus* and *An*. *gambiae* [55, 56].

## Conclusion

The genetic structure and variation in gene expression could be used to make an informed decision in future interventions especially as new insecticides are needed to control malaria across these countries. In addition, the finding of differences in the molecular basis of resistance of permethrin within a given country means that national resistance management strategies without characterization of underlying resistance mechanisms from localities may be flawed. The similarity of resistance profiles in Bulambuli, Lira, Tororo, and Kisumu, suggests that the same resistance management strategy could be implemented across these localities but might need to be different in Arua. These results have added to our understanding of the dynamics of vector species and will be valuable for planning effective vector control activities based on population genetic structure and resistance genes in Uganda and neighboring Kenya.

## Supporting information

S1 Table Loci and Primers sequences of *Anopheles funestus* microsatellite. (DOC)

## References

[1] WHO (2017) The world malaria report, vol. 2017 Geneva: World Health Organization.

[2] Okello PE, Van Bortel W, Byaruhanga AM, Correwyn A, Roelants P, et al (2006) Variation in malaria transmission intensity in seven sites throughout Uganda. The American journal of tropical medicine and hygiene 75: 219–225. 16896122

[3] Riveron JM, Ibrahim SS, Mulamba C, Djouaka R, Irving H, et al (2017) Genome-wide transcription and functional analyses reveal heterogeneous molecular mechanisms driving pyrethroids resistance in the major malaria vector Anopheles funestus across Africa. G3: Genes, Genomes, Genetics: g3 117.040147. 10.1534/g3.117.040147 28428243 PMC5473761

[4] Mulamba C, Riveron JM, Ibrahim SS, Irving H, Barnes KG, et al (2014) Widespread pyrethroid and DDT resistance in the major malaria vector Anopheles funestus in East Africa is driven by metabolic resistance mechanisms. PloS one 9: e110058 10.1371/journal.pone.0110058 25333491 PMC4198208

[5] Hemingway J, Ranson H (2000) Insecticide resistance in insect vectors of human disease. Annual review of entomology 45: 371–391. 10.1146/annurev.ento.45.1.371 10761582

[6] Morgan JC, Irving H, Okedi LM, Steven A, Wondji CS (2010) Pyrethroid resistance in an Anopheles funestus population from Uganda. PloS one 5: e11872 10.1371/journal.pone.0011872 20686697 PMC2912372

[7] Barnes KG, Weedall GD, Ndula M, Irving H, Mzihalowa T, et al (2017) Genomic footprints of selective sweeps from metabolic resistance to pyrethroids in African malaria vectors are driven by scale up of insecticide-based vector control. PLoS genetics 13: e1006539 10.1371/journal.pgen.1006539 28151952 PMC5289422

[8] Okoye PN, Brooke BD, Koekemoer LL, Hunt RH, Coetzee M (2008) Characterisation of DDT, pyrethroid and carbamate resistance in Anopheles funestus from Obuasi, Ghana. Transactions of the Royal Society of Tropical Medicine and Hygiene 102: 591–598. 10.1016/j.trstmh.2008.02.022 18405930

[9] Wondji CS, Coleman M, Kleinschmidt I, Mzilahowa T, Irving H, et al (2012) Impact of pyrethroid resistance on operational malaria control in Malawi. Proceedings of the National Academy of Sciences: 201217229 10.1073/pnas.1217229109 23118337 PMC3511128

[10] Riveron JM, Irving H, Ndula M, Barnes KG, Ibrahim SS, et al (2013) Directionally selected cytochrome P450 alleles are driving the spread of pyrethroid resistance in the major malaria vector Anopheles funestus. Proceedings of the National Academy of Sciences 110: 252–257. 10.1073/pnas.1216705110 23248325 PMC3538203

[11] Wondji CS, Morgan J, Coetzee M, Hunt RH, Steen K, et al (2007) Mapping a quantitative trait locus (QTL) conferring pyrethroid resistance in the African malaria vector Anopheles funestus. BMC genomics 8: 34 10.1186/1471-2164-8-34 17261170 PMC1790900

[12] Weedall GD, Mugenzi LM, Menze BD, Tchouakui M, Ibrahim SS, et al (2019) A cytochrome P450 allele confers pyrethroid resistance on a major African malaria vector, reducing insecticide-treated bednet efficacy. Science translational medicine 11: eaat7386 10.1126/scitranslmed.aat7386 30894503

[13] Mugenzi LM, Menze BD, Tchouakui M, Wondji MJ, Irving H, et al (2019) Cis-regulatory CYP6P9b P450 variants associated with loss of insecticide-treated bed net efficacy against Anopheles funestus. Nature communications 10: 1–11. 10.1038/s41467-018-07882-8 31604938 PMC6789023

[14] Wondji CS, Irving H, Morgan J, Lobo NF, Collins FH, et al (2009) Two duplicated P450 genes are associated with pyrethroid resistance in Anopheles funestus, a major malaria vector. Genome research. 10.1101/gr.087916.108 19196725 PMC2661802

[15] Amenya D, Naguran R, Lo TC, Ranson H, Spillings B, et al (2008) Over expression of a cytochrome P450 (CYP6P9) in a major African malaria vector, Anopheles funestus, resistant to pyrethroids. Insect molecular biology 17: 19–25. 10.1111/j.1365-2583.2008.00776.x 18237281

[16] Lukindu M, Bergey CM, Wiltshire RM, Small ST, Bourke BP, et al (2018) Spatio-temporal genetic structure of Anopheles gambiae in the Northwestern Lake Victoria Basin, Uganda: implications for genetic control trials in malaria endemic regions. Parasites & vectors 11: 246. 29661226 10.1186/s13071-018-2826-4 PMC5902950

[17] Lehmann T, Hawley W, Grebert H, Danga M, Atieli F, et al (1999) The Rift Valley complex as a barrier to gene flow for Anopheles gambiae in Kenya. Journal of Heredity 90: 613–621. 10589511 10.1093/jhered/90.6.613

[18] Lehmann T, Hawley WA, Kamau L, Fontenille D, Simard F, et al (1996) Genetic differentiation of Anopheles gambiae populations from East and West Africa: comparison of microsatellite and allozyme loci. Heredity 77: 192–200. 8760401 10.1038/hdy.1996.124

[19] Lehmann T, Licht M, Elissa N, Maega B, Chimumbwa J, et al (2003) Population structure of Anopheles gambiae in Africa. Journal of Heredity 94: 133–147. 10.1093/jhered/esg024 12721225

[20] Pinto J, Egyir-Yawson A, Vicente J, Gomes B, Santolamazza F, et al (2013) Geographic population structure of the African malaria vector Anopheles gambiae suggests a role for the forest-savannah biome transition as a barrier to gene flow. Evolutionary applications 6: 910 10.1111/eva.12075 24062800 PMC3779092

[21] Kaddumukasa MA, Wright J, Muleba M, Stevenson JC, Norris DE, et al (2020) Genetic differentiation and population structure of Anopheles funestus from Uganda and the southern African countries of Malawi, Mozambique, Zambia and Zimbabwe. Parasites & vectors 13: 1–13. 10.1186/s13071-020-3962-1 32070403 PMC7029513

[22] Gillies M, Coetzee M (1987) A supplement to the Anophelinae of Africa south of the Sahara (Afrotropical region).

[23] Koekemoer L, Kamau L, Hunt R, Coetzee M (2002) A cocktail polymerase chain reaction assay to identify members of the Anopheles funestus (Diptera: Culicidae) group. The American journal of tropical medicine and hygiene 66: 804–811. 10.4269/ajtmh.2002.66.804 12224596

[24] Organization WH (1998) Test procedures for insecticide resistance monitoring in malaria vectors, bio-efficacy and persistence of insecticides on treated surfaces: report of the WHO informal consultation, Geneva, 28–30 9 1998.

[25] Crawford JE, Guelbeogo WM, Sanou A, Traoré A, Vernick KD, et al (2010) De novo transcriptome sequencing in Anopheles funestus using Illumina RNA-seq technology. PloS one 5 10.1371/journal.pone.0014202 21151993 PMC2996306

[26] Gregory R, Darby AC, Irving H, Coulibaly MB, Hughes M, et al (2011) A de novo expression profiling of Anopheles funestus, malaria vector in Africa, using 454 pyrosequencing. PloS one 6. 10.1371/journal.pone.0017418 PMC3045460 21364769

[27] Irving H, Riveron J, Ibrahim S, Lobo N, Wondji C (2012) Positional cloning of rp2 QTL associates the P450 genes CYP6Z1, CYP6Z3 and CYP6M7 with pyrethroid resistance in the malaria vector Anopheles funestus. Heredity 109: 383–392. 10.1038/hdy.2012.53 22948188 PMC3499844

[28] Kwiatkowska RM, Platt N, Poupardin R, Irving H, Dabire RK, et al (2013) Dissecting the mechanisms responsible for the multiple insecticide resistance phenotype in Anopheles gambiae ss, M form, from Vallee du Kou, Burkina Faso. Gene 519: 98–106. 23380570 10.1016/j.gene.2013.01.036 PMC3611593

[29] Schmittgen TD, Livak KJ (2008) Analyzing real-time PCR data by the comparative C T method. Nature protocols 3: 1101 10.1038/nprot.2008.73 18546601

[30] Cohuet A, Dia I, Simard F, Raymond M, Fontenille D (2004) Population structure of the malaria vector Anopheles funestus in Senegal based on microsatellite and cytogenetic data. Insect molecular biology 13: 251–258. 10.1111/j.0962-1075.2004.00482.x 15157226

[31] Michel AP (2005) Population Genetic Structure of the Malaria Vector Anopheles Funestus: University Of Notre Dame. 10.1111/j.1365-294X.2005.02754.x 16313589

[32] Van Oosterhout C, Hutchinson WF, Wills DP, Shipley P (2004) MICRO‐CHECKER: software for identifying and correcting genotyping errors in microsatellite data. Molecular Ecology Notes 4: 535–538.

[33] Belkhir K (2004) 1996–2004 GENETIX 4.05, logiciel sous Windows TM pour la genetique des populations. http://wwwunivmontp2fr/~genetix/genetix/genetixhtm.

[34] Goudet J (1995) FSTAT version 2.9. 3.2. A program to estimate and test gene diversities and fixation indices Institute of Ecology, Lausanne, Switzerland.

[35] Raymond M (1995) GENEPOP (version 1.2): population genetics software for exact tests and ecumenicism. J Hered 86: 248–249.

[36] Wright S (1978) Evolution and the genetics of populations: a treatise in four volumes: Vol. 4: variability within and among natural populations: University of Chicago Press.

[37] Weir BS, Cockerham CC (1984) Estimating F‐statistics for the analysis of population structure. evolution 38: 1358–1370. 10.1111/j.1558-5646.1984.tb05657.x 28563791

[38] Pritchard JK, Stephens M, Donnelly P (2000) Inference of population structure using multilocus genotype data. Genetics 155: 945–959. 10835412 10.1093/genetics/155.2.945 PMC1461096

[39] Falush D, Stephens M, Pritchard JK (2003) Inference of population structure using multilocus genotype data: linked loci and correlated allele frequencies. Genetics 164: 1567–1587. 12930761 10.1093/genetics/164.4.1567 PMC1462648

[40] Fontaine MC, Baird SJ, Piry S, Ray N, Tolley KA, et al (2007) Rise of oceanographic barriers in continuous populations of a cetacean: the genetic structure of harbour porpoises in Old World waters. BMC biology 5: 30 10.1186/1741-7007-5-30 17651495 PMC1971045

[41] Evanno G, Regnaut S, Goudet J (2005) Detecting the number of clusters of individuals using the software STRUCTURE: a simulation study. Molecular ecology 14: 2611–2620. 10.1111/j.1365-294X.2005.02553.x 15969739

[42] Rousset F (1997) Genetic differentiation and estimation of gene flow from F-statistics under isolation by distance. Genetics 145: 1219–1228. 9093870 10.1093/genetics/145.4.1219 PMC1207888

[43] Holm S (1979) A simple sequentially rejective multiple test procedure. Scandinavian journal of statistics: 65–70.

[44] Gong Y, Li T, Zhang L, Gao X, Liu N (2013) Permethrin induction of multiple cytochrome P450 genes in insecticide resistant mosquitoes, Culex quinquefasciatus. International journal of biological sciences 9: 863 10.7150/ijbs.6744 24155662 PMC3805894

[45] Müller P, Warr E, Stevenson BJ, Pignatelli PM, Morgan JC, et al (2008) Field-caught permethrin-resistant Anopheles gambiae overexpress CYP6P3, a P450 that metabolises pyrethroids. PLoS genetics 4. 10.1371/journal.pgen.1000286 19043575 PMC2583951

[46] Riveron JM, Ibrahim SS, Chanda E, Mzilahowa T, Cuamba N, et al (2014) The highly polymorphic CYP6M7 cytochrome P450 gene partners with the directionally selected CYP6P9a and CYP6P9b genes to expand the pyrethroid resistance front in the malaria vector Anopheles funestus in Africa. BMC genomics 15: 817 10.1186/1471-2164-15-817 25261072 PMC4192331

[47] Barnes KG, Irving H, Chiumia M, Mzilahowa T, Coleman M, et al (2017) Restriction to gene flow is associated with changes in the molecular basis of pyrethroid resistance in the malaria vector Anopheles funestus. Proceedings of the National Academy of Sciences 114: 286–291. 10.1073/pnas.1615458114 28003461 PMC5240677

[48] Riveron JM, Osae M, Egyir-Yawson A, Irving H, Ibrahim SS, et al (2016) Multiple insecticide resistance in the major malaria vector Anopheles funestus in southern Ghana: implications for malaria control. Parasites & vectors 9: 504 10.1186/s13071-016-1787-8 27628765 PMC5024453

[49] Vontas J, Grigoraki L, Morgan J, Tsakireli D, Fuseini G, et al (2018) Rapid selection of a pyrethroid metabolic enzyme CYP9K1 by operational malaria control activities. Proceedings of the National Academy of Sciences 115: 4619–4624. 10.1073/pnas.1719663115 29674455 PMC5939083

[50] Besansky NJ, Lehmann T, Fahey GT, Fontenille D, Braack LE, et al (1997) Patterns of mitochondrial variation within and between African malaria vectors, Anopheles gambiae and An. arabiensis, suggest extensive gene flow. Genetics 147: 1817–1828. 9409838 10.1093/genetics/147.4.1817 PMC1208348

[51] Michel AP, Grushko O, Guelbeogo WM, Lobo NF, Sagnon NF, et al (2006) Divergence with gene flow in Anopheles funestus from the Sudan Savanna of Burkina Faso, West Africa. Genetics. 10.1534/genetics.106.059667 16648581 PMC1526678

[52] Cohuet A, Dia I, Simard F, Raymond M, Rousset F, et al (2005) Gene flow between chromosomal forms of the malaria vector Anopheles funestus in Cameroon, Central Africa, and its relevance in malaria fighting. Genetics 169: 301–311. 10.1534/genetics.103.025031 15677749 PMC1448888

[53] Ayala D, Caro-Riaño H, Dujardin J-P, Rahola N, Simard F, et al (2011) Chromosomal and environmental determinants of morphometric variation in natural populations of the malaria vector Anopheles funestus in Cameroon. Infection, Genetics and Evolution 11: 940–947. 10.1016/j.meegid.2011.03.003 21414420 PMC3665408

[54] Samb B, Dia I, Konate L, Ayala D, Fontenille D, et al (2012) Population genetic structure of the malaria vector Anopheles funestus, in a recently re-colonized area of the Senegal River basin and human-induced environmental changes. Parasites & vectors 5: 188. 22950576 10.1186/1756-3305-5-188 PMC3503558

[55] Chen H, Minakawa N, Beier J, Yan G (2004) Population genetic structure of Anopheles gambiae mosquitoes on Lake Victoria islands, west Kenya. Malaria journal 3: 48 10.1186/1475-2875-3-48 15581429 PMC543573

[56] Ayala D, Le Goff G, Robert V, de Jong P, Takken W (2006) Population structure of the malaria vector Anopheles funestus (Diptera: Culicidae) in Madagascar and Comoros. Acta tropica 97: 292–300. 10.1016/j.actatropica.2005.12.002 16464433

