# Supplementary material for: A differential expression of pyrethroid resistance genes in the malaria vector Anopheles funestus across Uganda is associated with patterns of gene flow
Source: PLoS One. 2020 Nov 10;15(11):e0240743. doi: 10.1371/journal.pone.0240743 (PMC7654797; doi:10.1371/journal.pone.0240743)
Supplement: S1 Table — (DOC) [file pone.0240743.s001.doc]

| **Locus** | **Primers sequences (5′-3′)** | |
| --- | --- | --- |
| FUNL F | | AACAGTGGAAGGCAAATTGC |
| FUNL R | | GCACGGTTACCACTGCTCA |
| AFUB10 F | | TGTCCATGTACAACCGCAAC |
| AFUB10 R | | TTCTCCAGCATCATCAGCAC |
| AFUB11 F | | CAGTTTCTGCGTGGAGGAAT |
| AFUB11 R | | AGCAGCTGATGAGCCATCTC |
| AFND5 F | | TTTGATCGACGGACTAGTGTGT |
| AFND5 R | | GGTTTGATGGGTGGAAAC |
| FUNO F | | GCACACATTTCAGGCAGC |
| FUNO R | | GCCCACATTCTGCACCTT |
| AFUB3 F | | GGGAAGGATTCGACCTTAGC |
| AFUB3 R | | GCCGCCATTTAGTAGCAGTT |
| FUNF F | | GCCTTCAGTTTCGATTGGCG |
| FUNF R | | AATAAGATGCGACCGTGGC |
| AFUB12 F | | TGGGGAACTGGTCGTTAGAG |
| AFUB12 R | | CTGGTGATGGGATTGAGGAT |
| AFND19 F | | GCAAGCTGTACGCAGAGAG |
| AFND19 R | | ATCGATGGGAGTTATTATACGC |
| FUNQ F | | GCAAACTGCTAGTAAATGTTTCC |
| FUNQ R | | ACATTTCCACAATTTGCGC |
| AFND40 F | | GTTCTCCATCGCTGTTCTACTC |
| AFND40 R | | TATAACGTTTCGTACACACGCC |
| AFUB6 F | | GCTTCTTCTCCCCTAATCTG |
| AFUB6 R | | TCCTGCTTTTTAGTTTGTCG |
| FUNR F | | TTATGAACAGCACTGTAGCA |
| FUNR R | | GTCTCATCTATTCACTTTCCG |
| AFND6 F | | CGATGCCCACTAATTCAAG |
| AFND6 R | | GCCACCCAAAGTATAGTGAAG |
| AFND30 F | | AAAGCGCACTTTATGAACG |
| AFND30 R | | AAAACAAACACAGGAAGGC |
| AFND32 F | | CCGCACACCAACTTACACTC |
| AFND32 R | | TGGCGTGGGATTAAATAGG |
| AFND7 F | | CGAATTCATCTCGTAGAGACC |
| AFND7 R | | TAATACACACGCTTCGCTTC |

**S1 Table: Loci and Primers sequences of *Anopheles funestus* microsatellite**

|  |
| --- |
|  |
|  |
|  |
|  |
|  |
|  |
|  |
